# Stress-Relaxation and Cyclic Behavior of Human Carotid Plaque Tissue

**DOI:** 10.3389/fbioe.2020.00060

**Published:** 2020-02-11

**Authors:** Phani Kumari Paritala, Prasad K. D. V. Yarlagadda, Rhys Kansky, Jiaqiu Wang, Jessica Benitez Mendieta, YuanTong Gu, Tim McGahan, Thomas Lloyd, Zhiyong Li

**Affiliations:** ^1^School of Mechanical, Medical and Process Engineering, Queensland University of Technology, Brisbane, QLD, Australia; ^2^Department of Vascular Surgery, Princess Alexandra Hospital, Brisbane, QLD, Australia; ^3^Department of Radiology, Princess Alexandra Hospital, Brisbane, QLD, Australia

**Keywords:** carotid plaque, mechanical behavior, tensile test, stress-relaxation test, cyclic test

## Abstract

Atherosclerotic plaque rupture is a catastrophic event that contributes to mortality and long-term disability. A better understanding of the plaque mechanical behavior is essential for the identification of vulnerable plaques pre-rupture. Plaque is subjected to a natural dynamic mechanical environment under hemodynamic loading. Therefore, it is important to understand the mechanical response of plaque tissue under cyclic loading conditions. Moreover, experimental data of such mechanical properties are fundamental for more clinically relevant biomechanical modeling and numerical simulations for risk stratification. This study aims to experimentally and numerically characterize the stress-relaxation and cyclic mechanical behavior of carotid plaque tissue. Instron microtester equipped with a custom-developed setup was used for the experiments. Carotid plaque samples excised at endarterectomy were subjected to uniaxial tensile, stress-relaxation, and cyclic loading protocols. Thirty percent of the underlying load level obtained from the uniaxial tensile test results was used to determine the change in mechanical properties of the tissue over time under a controlled testing environment (Control tests). The stress-relaxation test data was used to calibrate the hyperelastic (neo-Hookean, Ogden, Yeoh) and linear viscoelastic (Prony series) material parameters. The normalized relaxation force increased initially and slowly stabilized toward the end of relaxation phase, highlighting the viscoelastic behavior. During the cyclic tests, there was a decrease in the peak force as a function of the cycle number indicating mechanical distension due to repeated loading that varied with different frequencies. The material also accumulated residual deformation, which increased with the cycle number. This trend showed softening behavior of the samples. The results of this preliminary study provide an enhanced understanding of *in vivo* stress-relaxation and cyclic behavior of the human atherosclerotic plaque tissue.

## Introduction

Atherosclerosis is a chronic inflammatory disease characterized by hardening or narrowing of the arteries (Ross, 1999). It occurs due to the build-up of plaque, which is often asymptomatic. Carotid artery disease is responsible for 25% of ischemic strokes (Levy et al., 2008) occurring due to plaque rupture in lesions characterized by a thin fibrous cap covering a large necrotic core (Virmani et al., 2000; Narula et al., 2008; Finn et al., 2010). Luminal stenosis is a key feature of atherosclerosis and is traditionally assessed using angiographic techniques. However, the majority of the plaques that cause acute events are not highly stenotic, because the artery undergoes positive remodeling before the plaque encroaches into the lumen (Glagov et al., 1987). Moreover, plaque content can be considered more critical than plaque size (Shah, 2003; Corti et al., 2004) and is responsible for life-threatening events. Therefore, various imaging modalities such as ultrasound and high-resolution magnetic resonance imaging (MRI) (Trivedi et al., 2008; Vancraeynest et al., 2011; Makris et al., 2015) are used to identify the morphological features of the atherosclerotic plaque. Despite advancements in treatment and management procedures, stroke remains the leading cause of mortality and long-term morbidity in developed countries (Benjamin et al., 2018).

Plaque disruption precedes most acute events and occurs when the stresses developed exceeds the mechanical strength of the fibrous cap (Li et al., 2006a, b). Additionally, the initiation and progression of atherosclerosis is a complex interaction between the systemic risk factors, hemodynamics, and vascular biology (Hsieh et al., 2014; Morbiducci et al., 2016). Therefore, it was suggested that biomechanical analysis of atherosclerotic plaque rupture, involving various techniques such as structural only, fluid-structure interaction and fatigue models based on patient-specific geometries of carotid arteries may assist in predicting the vulnerability and risk stratification of the plaques (Tang et al., 2005; Gao et al., 2011; Teng et al., 2012; Holzapfel et al., 2014; Huang et al., 2014; Lu et al., 2015; Paritala et al., 2018; Wang et al., 2019).

Biomechanical properties of the atherosclerotic plaque tissue are among the critical inputs for accurate numerical simulations and clinical applicability. Several studies have identified the mechanical behavior of peripheral plaque tissue using tensile tests, compressive tests and fracture toughness tests under static loads (Holzapfel et al., 2004; Barrett et al., 2009, 2016b; Ebenstein et al., 2009; Maher et al., 2009; Lawlor et al., 2011; Chai et al., 2013; Mulvihill et al., 2013; Teng et al., 2014; Cunnane et al., 2015, 2016a,b). Under the fatigue environment of the cardiovascular system, carotid arteries undergo large deformations due to pulsatile blood flow that can often cause atherosclerotic plaque rupture (Bank et al., 2000; Pei et al., 2013; Paritala et al., 2018). Moreover, biological tissues exhibit time-dependent properties. Therefore, understanding the mechanical response of the plaque tissue using stress-relaxation and cyclic stretching is beneficial in designing disease treatment and management procedures. However, investigations that characterize the mechanical behavior of carotid plaques under cyclic loading conditions and the viscoelastic properties remains relatively unexplored, except a few studies on arterial elastin, porcine veins and arteries, abdominal aortic aneurysm, ligaments, muscle and skin tissues (Fung, 1967; Salunke et al., 2001; Toms et al., 2002; Sarver et al., 2003; Gasser et al., 2008; Heiland et al., 2013; Pasquesi et al., 2016, Pasquesi and Margulies, 2017; Remache et al., 2018; Wang et al., 2018; Yoo et al., 2009; Zou and Zhang, 2011).

Since limited information is available regarding the stress-relaxation and cyclic behavior of the carotid plaque tissue, the primary goal of this study was to enhance the understanding of the mechanical behavior of plaque tissue under the cyclic cardiac environment. Therefore, in this study, we investigated the effect of cyclic loading and the stress-relaxation on the mechanical behavior of the carotid plaque tissue. Initially, uniaxial tensile tests and control tests were performed to determine the ultimate strength and change in elastic properties of the tissue under a controlled testing environment. Subsequently, stress-relaxation and cyclic tests were performed. Experimental data regarding the stress-relaxation test was used to determine the hyperelastic and viscoelastic material properties.

## Materials and Methods

### Sample Acquisition and Storage

**FIGURE 1:**
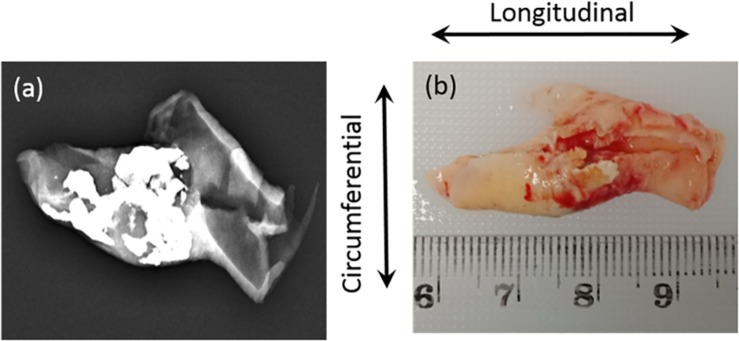
**(a)** Low energy X-ray used to identify the calcium content; **(b)** Sample collected from hospital with cutting direction (circumferential).

Ten patients with carotid stenosis >70%, who were scheduled for carotid endarterectomy in the Princess Alexandra Hospital (PAH), were recruited for this study. This study was approved by the Metro South Human Research Ethics Committee (HREC/17/QPAH/181), and patient consent forms were obtained. Patient demographic data (see Supplementary Table S1) and carotid plaque tissue samples were collected following carotid endarterectomy. This method of acquisition resulted in all samples being cut longitudinally. Immediately after excision, the plaque samples were placed in phosphate buffered saline (PBS) and in a container with ice for preservation. The samples were taken for scanning in a low energy X-ray using a low-dose mammography machine (Mammomat Inspiration with Prime Technology, Siemens, New York, United States) at PAH. The scanning was operated at 23 kVp and 14 mAs with no compression and a pixel resolution of 85 μm. After a low energy X-ray (Figure 1a), the samples were transported to the biomechanical engineering facility. There the samples were cut into 3–4 mm sections in the circumferential direction indicated by an arrow in Figure 1b. The cut sections were then placed in individual Eppendorf tubes and frozen using dry ice. These Eppendorf tubes were then stored at −20°C within 2 h of excision. This method of storage was preferred as it has been shown not to have a significant effect on the mechanical properties of arterial tissue (Ebenstein et al., 2009; O’Leary et al., 2014).

### Specimen Preparation

**FIGURE 2:**
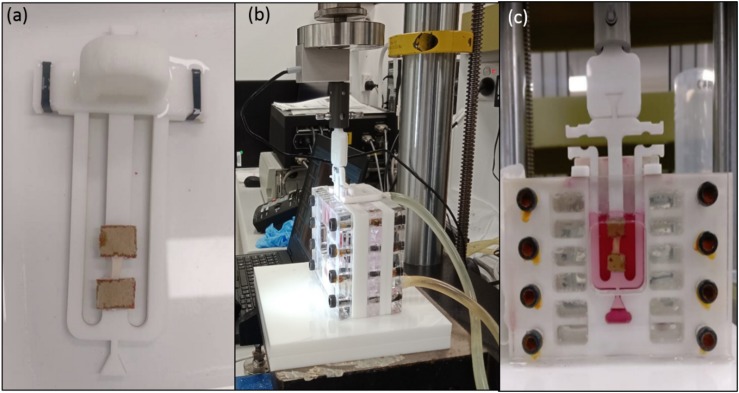
**(a)** Sample fixed to the custom designed frame with sandpaper and LOCTITE glue; **(b)** Experimental setup showing tubes connected to the hot water bath to maintain physiological temperature; **(c)** Sample hydration was maintained using DMEM supplemented with 20% fetal bovine serum (FBS), 50 μg/ml L-ascorbic acid and 50 IU/mL Penicillin-Streptomycin.

On the day of testing, the tissue samples were thawed for an hour at 4°C and subsequently immersed in PBS at room temperature for 15 min. If multiple tissue segments were tested on a day, segments were removed individually to minimize any potential variance. The specimens were cut in the circumferential direction (perpendicular to blood flow) to a length of 11.33 ± 1.16 mm and width of 3.14 ± 0.47 mm using a surgical blade. Strip thickness ranged from 1.17 ± 0.23 mm and varied along the length of the tissue depending on disease severity. In total, 16 (25) tensile, 5 (6) control, 8 (10) stress relaxation, and 8 (11) cyclic strips were tested successfully (total number of samples tested in brackets). Once the strips were prepared, they were fixed to custom designed frames using pieces of sandpaper and super-adhesive water-resistant glue (LOCTITE) (Figure 2a). PBS was sprayed onto the samples during the preparation phase to ensure that they remained hydrated. Later, the strip was uniquely labeled and placed in PBS until further mechanical testing. The gauge length of the samples being tested was 5 mm. The width to length ratio of samples was 0.63 ± 0.09 which is between 0.1 and 1 and therefore all samples were suitable for tensile testing (Mulvihill and Walsh, 2013; Walsh et al., 2014).

### Experimental Procedure and Testing Protocols

An Instron 5848 MicroTester (Instron Corporation, Norwood, MA, United States) with a load cell of 5N was used to conduct all tests. A custom-developed setup was used to mount the sample, maintain sample hydration and physiological temperature as shown in Figures 2b,c. For the uniaxial tensile tests sample hydration was maintained using PBS followed by Dulbecco’s modified eagle’s medium (DMEM) supplemented with 20% fetal bovine serum (FBS), 50 μg/ml L-ascorbic acid and 50 IU/mL Penicillin-Streptomycin for the control, stress-relaxation and cyclic tests (Gasser et al., 2008). To mimic *in vivo* conditions during testing, an in-house developed setup was connected to a circulating water bath maintained at 37 ± 1°C. All experiments were conducted in a displacement-controlled mode, and the data were recorded using WaveMaker- Editor 9.2.00 software (Instron Corporation, Norwood, MA, United States). Once the samples were carefully secured to the frame following the specimen preparation, a preload of 0.1 N was applied to remove any slack of the sample. Following this, the load cell was balanced before performing each test.

#### Uniaxial Tensile Test

**FIGURE 3:**
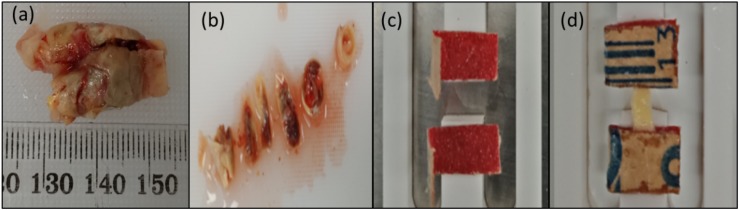
Preparation of test specimens: **(a)** Carotid plaque sample collected from the hospital; **(b)** Representative segments cut from the plaque tissue sample; **(c)** Sandpaper glued to the frame; **(d)** Sample glued to the sandpaper.

Uniaxial tensile test was performed to investigate the tissue’s ultimate strength (F_ult_). Quasi-static loading conditions was used to characterize the mechanical behavior of the tissue (Holzapfel et al., 2004). The samples were elongated at a rate of 0.1 mm/s until failure. In total, 25 strip sections obtained from 4 patients were tested. The number of strips cut from each patient depended on the size of the sample recovered from the surgery. Some sections were omitted from the tests due to the visibility of cracks and high variability in the thickness. Out of 25 strips, the data for 16 strips were used to calculate the ultimate strength. The data from the remaining samples were omitted from the analysis because the tissue slipped or tore at the sandpaper. Figure 3 shows the sample, specimen preparation, and insertion into frame.

The recorded load-displacement data was used to compute the stretch ratio and Cauchy stress (Walsh et al., 2014; Teng et al., 2015) using Eqs 1 and 2. The peak load adjacent (F_ult_) to the sudden or steep decline of the load-displacement curve was identified for each sample being tested. An underlying load level of 0.3F_ult_ was calculated so that the value remains below the elastic limit. The displacement corresponding to the 0.3F_ult_ was identified for each strip. A value below the average displacement was used as the input for the control tests performed under cyclic tension (displacement-controlled experiments).

(1) $$\lambda =\frac{L}{L_{o}}$$

(2) $$\sigma =\lambda \,\frac{F}{A_{o}}$$

Where σ = Cauchy stress; λ = Stretch Ratio;

*F* = Applied force; *A*_o_ = Original Area

*L*_o_ = Gauge length; L = Deformed length

#### Control Tests

Control tests were performed to investigate the change in elastic properties of the tissue over time in the controlled testing environment. Following sample preparation and insertion into the frame the specimen was tested every 6 h over 24 h. Between each test the sample was left in an unloaded state. The test specimen was subjected to nine cyclic tension cycles at a rate of 0.2 mm/s. An average displacement value (considering the heterogeneity of the samples an average value of displacement was used as amplitude) corresponding to 0.3F_ult_ of the samples tested (calculated from the uniaxial tests) was applied for each tension cycle (Gasser et al., 2008). A total of six strips from two patients were tested, out of which one was omitted from the analysis due to an error in recording the data. The data for the last three cycles were analyzed to calculate the elastic properties of the samples tested at 0, 6, 12, 18, and 24 h of testing.

The data corresponding to the last three cycles of the control tests was used to evaluate the relative elastic stiffness of the samples being tested for a prolonged duration of time. A third-degree polynomial Eq. 3 were used to quantify the stiffness of the carotid plaque sample at 0, 6, 12, 18, and 24 h. Eqs. 4 and 5 were used to quantify the relative stiffness of the sample (Gasser et al., 2008).

(3) $$f_{i}=P_{1}\,x^{3}+P_{2}\,x^{2}+P_{3}\,x+P_{4}$$

(4) $$\delta_{i}=\frac{{\bar{S}}_{i}}{{\bar{S}}_{0}}$$

(5) $${\bar{S}}_{i}=\frac{1}{u_{u\,l\,t}}\,\int_{0}^{u_{u\,l\,t}}\left(\frac{d\,f_{i}}{d\,u}\right)\,d\,u=\frac{1}{u_{u\,l\,t}}\,\left(f_{i}\,\left(u_{u\,l\,t}\right)-f_{i}\,\left(0\right)\right)$$

Where i = 0, 6, 12, 18, 24;

δ_i_ = relative specimen stiffness

*u*_ult_ = displacement corresponding to the ultimate load F_ult_

${\bar{S}}_{\text{i}}$ = average stiffness at time = i

${\bar{S}}_{0}$ = average stiffness at time = 0

#### Stress-Relaxation Test Profile

**FIGURE 4:**
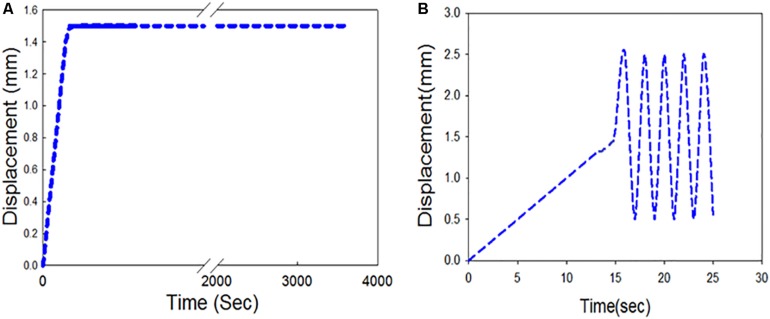
Displacement controlled profiles **(A)** Stress-relaxation loading protocol; **(B)** Cyclic test loading protocol.

Stress-relaxation tests were performed to understand the relaxation behavior of the carotid plaque tissue. The initial gauge length was 5 mm for all strips. Each strip was stretched up to 30% of the gauge length at a rate of 0.1 mm/s (Profile 1) or 1 mm/s (Profile 2) under displacement control mode to investigate the effects of strain rate on stress-relaxation. A total of 8 strips were tested from four patients, with two strips from each patient. For each patient, one of their strips was subjected to Profile 1, while the other specimen from the same patient was subjected to Profile 2 to minimize variability between the profiles. Stress-relaxation test profile is shown in Figure 4A. Load, position, and time data were recorded by the Instron testing software. Detailed information about the test profiles is presented in of the Supplementary Table S2.

Recorded load and displacement data were analyzed to quantify the changes in mechanical properties over time (Remache et al., 2018). Eight carotid plaque strips were subjected to a stress-relaxation test at a strain rate of 0.1 and 1 mm/s (4 samples each). Instantaneous variation in the force *F*(*t*) with respect to the force at the beginning of stress-relaxation *F*(*t*_0_) was calculated. The variation due to the samples being collected from different patients was normalized by dividing it by the force at the beginning of each test. The normalized variation in the force [*F*_SR_(*t*)] as a function of the relaxation time is thus given by the following relationship:

(6) $$F_{\mathrm{SR}}\,\left(t\right)=\frac{F\,\left(t_{0}\right)-F\,\left(t\right)}{F\,\left(t_{0}\right)}$$

The material constants were identified using ABAQUS/Explicit 6.13 (Dassault Systèms, SIMULIA Corp., Johnston, RI, United States) calibration tool. The stress response of the plaque tissue was assumed to be incompressible, isotropic, and homogenous (Holzapfel, 2000). Non-linear isotropic modeling and linear isotropic viscoelastic modeling were separately used to calibrate the stress-relaxation test data. Neo-Hookean, Ogden (order 1) and Yeoh models were used to calibrate the non-linear portion of the test data, and a linear isotropic viscoelastic model which is represented in terms of Prony series was used to calibrate the viscoelastic portion of the test data. The nominal stress-strain values were calculated from the force-displacement data. Later the data that corresponded to the initial displacement of the sample before reaching 30%-gauge length was used to calibrate the non-linear behavior. Subsequently, the normalized relaxation phase data was used to calibrate the linear viscoelastic behavior (see Supplementary Figure S1).

The strain energy density function for an incompressible isotropic Neo-Hookean model is expressed as

(7) $$W=C_{10}\,\left(I_{1}-3\right)$$

Where *C*_*10*_ is the material constant, *I*_*1*_ is the first invariant of Cauchy-Green tensor ($I_{1}=\lambda_{1}^{2}+\lambda_{2}^{2}+\lambda_{3}^{2}$, where λ_i_ are the principal stretches). The strain energy density function for Ogden material model (Ogden, 1972) is expressed as:

(8) $$W=\sum_{i=1}^{N}\frac{\mu_{i}}{\alpha_{i}^{2}}\,\left(\lambda_{1}^{\alpha_{i}}+\lambda_{2}^{\alpha_{i}}+\lambda_{3}^{\alpha_{i}}-3\right)$$

Where λ_i_ (where *i* = 1, 2, 3) are the principal stretches; N is a positive integer and α_i,_ and μ_i_ are the material constants. The strain energy density function for Yeoh material model is expressed as

(9) $$W=\sum_{i=1}^{3}C_{10}\,{\left(I_{1}-3\right)}^{i}$$

Where *C*_*10*_ is the material constant, *I*_*1*_ is the first invariant of Cauchy-Green tensor. To study the time-dependent mechanical behavior of carotid plaque tissue for load relaxation in displacement control, a linear isotropic viscoelastic model represented in terms of Prony series is given by:

(10) $$G\,\left(t\right)=G_{\infty }+\sum_{i=1}^{N_{G}}g_{i}\,\exp\,\left(-t/\tau_{i}\right)$$

Where *G*_∞_ is the long-term modulus once the material is totally relaxed, *g*_i_ and τ_i_ are material constants.

#### Cyclic Test Profile

Cyclic tests were performed to understand the mechanical behavior of the plaque tissue under continuous cyclic loading. The gauge length remained at 5 mm, and each of the strips were exposed to a displacement controlled relative ramp of up to 30% of the gauge length (+1.5 mm) at 0.1 mm/s. A sinusoidal waveform (Figure 4B) was then applied to fluctuate between 10 and 50% stretch ratio (±1 mm) for 2 h. A total of 8 strips were tested from four patients, with two strips from each patient one being subjected to a sinusoidal frequency of 1 Hz and the other subjected to a frequency of 1.5 Hz. These frequencies were used as they are above and below the physiological average frequency of heartbeat (∼1.2 Hz). The number of cycles in a 2-hour duration for 1 Hz frequency was 7200 and for 1.5 Hz was 10,800 cycles. Different profiles were created to evaluate the effects of different frequencies on cyclic activity. Detailed information about the test profiles is presented in the Supplementary Table S3.

The variation in the peak force for each cycle *F*_max_(*c*_n_) with respect to the peak force corresponding to the first cycle *F*_max_(*c*_1_) was calculated from the load displacement data recorded. To normalize the variation, this was then divided by the peak force corresponding to the first cycle as represented in Eq. 11.

(11) $${\mathrm{PF}}_{c}=\frac{F_{\max}\,\left(c_{1}\right)-F_{\max}\,\left(c_{n}\right)}{F_{\max}\,\left(c_{1}\right)}$$

Tangent modulus were calculated from the tangent at the beginning of the unloading curve of each cycle. Variation in tangent modulus (T) of the carotid plaque corresponding to the first cycle *TM*(*c*_1_) and any cycle *n TM*(*c*_*n*_)was calculated to quantify the changes in mechanical properties under cyclic test (Eq. 12).

(12) $$T=\frac{\mathrm{TM}\,\left(c_{1}\right)-\mathrm{TM}\,\left(c_{n}\right)}{\mathrm{TM}\,\left(c_{1}\right)}$$

Residual strain (RS) accumulation of the carotid plaque tissue was calculated as a function of cycle number using the following relationship:

(13) $$\mathrm{RS}=\frac{D\,\left(c_{n}\right)-D\,\left(c_{2}\right)}{L_{0}}$$

Where the gauge length is L_0_, and D(c_2_) and D(c_n_) are the maximum deformations corresponding to zero forces in the second cycle and any cycle *n*, respectively.

## Results

### Uniaxial Tensile Tests

**FIGURE 5:**
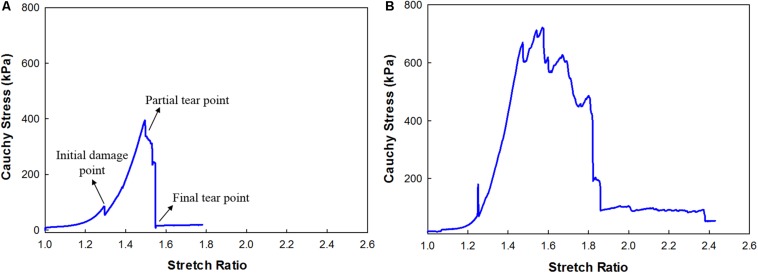
Cauchy Stress and stretch response of the carotid plaque strip **(A)** without calcification; **(B)** with calcification.

In total, 16 strips cut from 4 patients were analyzed for Cauchy stress and extensibility. Data corresponding to the tissue samples that slid or tore near the sandpaper was excluded. The width and thickness of the strips included for analysis were 3 (2) mm and 1.10 (0.7) mm [Median (Range)]. The gauge length was maintained at 5 mm. The percentage area of calcification was measured from the low energy x-ray of the carotid plaque tissues using ImageJ^1^ (Supplementary Figure S2) and the values are tabulated in Table 1. The samples were classified based on the % calcification area content 0–10% (low calcification content−soft), 11–20% (medium calcification content−mixed) and 21% and above (high calcification content−hard). Figure 5 shows the load-displacement curve for the tissue with and without calcification. The samples with high calcification content have higher stress values. Also, a sudden drop in the stress values has been observed at different points during the test when there is a separation between calcification and the surrounding tissue. The median of the stretch ratio (range) for all samples at failure was 1.56 (1.50). Mean Cauchy stress values for all the strips tested was 683.16 (2214.33) kPa. The stretch ratio and Cauchy stress values for strips with no-calcification was 1.57 (0.15) mm, 322.48 (528.98) kPa, while for strips with calcification was 1.79 (1.5) mm, 888.75 (1724.13) kPa (Table 2).

**TABLE 1:** Stratification of the carotid plaque tissue based on % calcification from low energy X-ray. TA, Total area; CA, Calcification Area; TI, T indicates sample used for Tensile test; I, indicates patient number; S1, S indicates Stress-relaxation/Fatigue test sample 1 indicates Patient number 1.

ID	TA	CA	% Cal	Content
**Tensile test samples**				
TI	32.	7.35	22.97	High
TII	40.32	0.30	0.75	Low
TIII	50.07	5.91	11.8	Medium
TIV	74.62	12.45	16.47	Medium
**Stress-relaxation and cyclic test samples**				
S1	55.34	0.99	1.79	Low
S2	43.59	2.19	5.04	Low
S3	255.9	74.59	29.14	High
S4	338.34	65.55	19.37	Medium

**TABLE 2:** Dimensions, width to gauge length ratio, Cauchy stress, the stretch ratio at failure and type of the strip [Type: Soft-low calcification content (0 – 10%), Medium- medium calcification content (11 – 20%), Hard- high calcification content (21% and above) as observed from low energy X-ray].

ID	Length (mm)	Width (mm)	Thickness (mm)	Width/Gauge length ratio	Cauchy Stress (kPa)	Stretch ratio at failure	Type
TI (a)	10	2	1.1	0.4:1	271.80	1.60	Soft
TI(b)	10	3	1.5	0.6:1	555.30	1.47	Mixed
TI(c)	10	4	1.5	0.8:1	654.37	1.91	Hard
TII(b)	9	3	0.8	0.6:1	711.95	1.82	Mixed
TII(c)	10	3	0.8	0.6:1	594.06	1.86	Mixed
TII(d)	10	3	1	0.6:1	322.48	1.64	Soft
TII(e)	10	3	1	0.6:1	594.08	1.57	Soft
TIII(a)	10	3	1.1	0.6:1	2279.43	2.96	Mixed
TIII(b)	10	3	1.1	0.6:1	390.35	1.49	Soft
TIV(a)	10	2.5	1	0.5:1	717.78	1.57	Mixed
TIV(b)	10	2	0.8	0.4:1	1225.09	1.48	Mixed
TIV(c)	11	3	1.1	0.6:1	1705.09	1.82	Mixed
TIV(d)	12	3	1.3	0.6:1	1010.97	1.47	Hard
TIV(e)	11	3	1.1	0.6:1	1258.00	1.79	Hard
TIV(f)	11	3	1.2	0.6:1	888.75	1.51	Mixed
TIV(g)	12	3.1	1	0.62:1	65.10	1.54	Soft

### Control Tests

**FIGURE 6:**
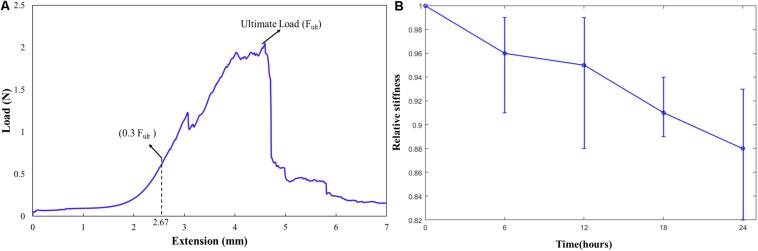
**(A)** A typical curve representing the ultimate load (F_ult_), 0.3F_ult_ and the corresponding displacement; **(B)** Relative stiffness of the carotid plaque samples over time in supplemented DMEM at 37°C.

In total, five strips cut from 2 patients were analyzed for the change in elastic properties when tested in DMEM maintained at 37°C for 24 h. A typical curve representing ultimate load (F_ult_), 0.3F_ult_ and the corresponding displacement is shown in Figure 6A. An average displacement value calculated corresponding to 0.3F_ult_ load was given as input displacement for the nine tension cycles in the control test. The values of the polynomial coefficients and relative specimen stiffness of the samples tested [calculated from Eqs. (3–5)] were used to investigate the change in the elastic properties of the tissue over time. It was found that the sample elastic stiffness decreased over time, and the maximum change was 8.7% within 6 h, as shown in Figure 6B. It should be noted that in this study cyclic tests were conducted for only 2 h.

### Stress-Relaxation Tests

**FIGURE 7:**
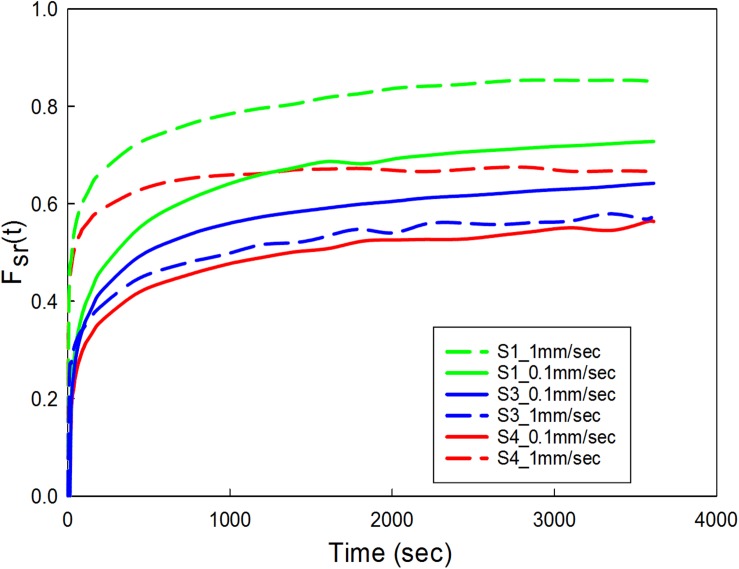
Carotid plaque tissue was subjected to 30% stretch at 0.1 and 1 mm/s strain rate. The stretch was maintained for 1 h. Normalized variation in force as a function of time was represented for three samples (S1, S3, and S4) at two different strain rates. S1-low calcification, S3-high calcification, S4- medium calcification.

**FIGURE 8:**
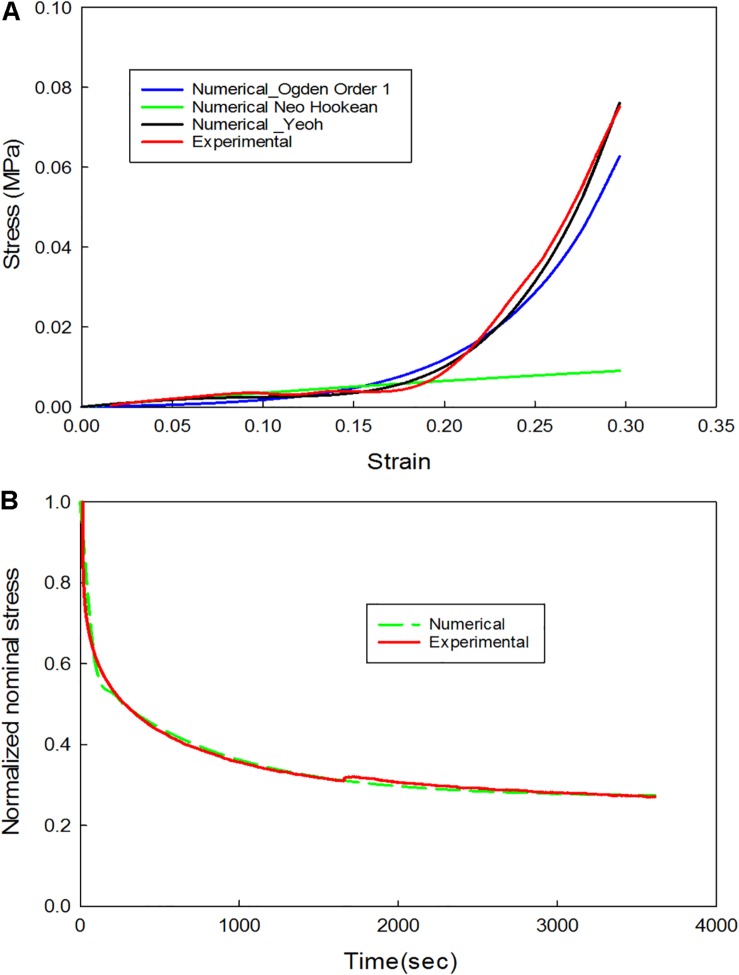
**(A)** Comparison of experimental and numerical results for the non-linear behavior (extension portion of stress-relaxation test data); **(B)** Comparison of experimental and numerical results for the linear viscoelastic behavior (stress-relaxation portion of the stress-relaxation test data).

The data corresponding to the dimension, width to gauge length ratio and type of the strip is listed in Table 3. The normalized variation in force *F*_SR_(*t*) is different for different strain rates with larger values for 1 mm/s and are represented in Figure 7. Figures 8A,B compare the experimental and numerical results of the stress-relaxation data. Material coefficients associated with neo-Hookean, Ogden (Order 1) [hyperelastic behavior], Yeoh models and, Prony series [viscoelastic behavior] are identified for each strip tested and listed in Table 4. Significant variability and non-linear behavior of the samples were observed.

**TABLE 3:** Gauge length, width, width- gauge length ratio, type for each strip tested [Type: Soft- low calcification content (0 – 10%), Medium- medium calcification content (11 – 20%), Hard- high calcification content (21% and above) as observed from low energy X-ray].

ID	Length (mm)	Width (mm)	Thickness (mm)	Width/Gauge length ratio	Type
S1(a)	13	4	1.5	0.8:1	Mixed
S1(b)	12	3.5	1	0.7:1	Soft
S2(a)	13	3.5	1.5	0.7:1	Mixed
S2(b)	12	3	1	0.6:1	Mixed
S3(a)	12	3.5	1.2	0.7:1	Hard
S3(b)	12	3.5	1	0.7:1	Mixed
S4(a)	12	3	1	0.6:1	Soft
S4(b)	12	3	1.2	0.6:1	Mixed
S1(c)	12	3.5	1.5	0.7:1	Mixed
S1(d)	13	4	1.5	0.8:1	Mixed
S2(c)	13	3	1.2	0.6:1	Soft
S2(d)	12	3.5	1	0.7:1	Mixed
S3(c)	12	3	1.2	0.6:1	Mixed
S3(d)	13	3.5	1.5	0.7:1	Hard
S4(c)	12	3.5	1.5	0.7:1	Hard
S4(d)	12	3.6	1.5	0.72:1	Mixed

**TABLE 4:** Material parameters identified from the experimental data (Hyperelastic and linear Viscoelastic).

Sample		S1_0.1 mm/s	S1_1 mm/s	S2_0.1 mm/s	S2_1 mm/s	S3_0.1 mm/	S3_1 mm/	S4_0.1 mm/s	S4_1 mm/s
**Material Model**									
Prony series	g1	0.41	0.46	0.338	0.45	0.39	0.34	0.34	0.51
	τ_*1*_	44.56	4.35	20.51	6.36	50.39	9.79	46.72	4.28
	g2	0.32	0.17	0.23	0.27	0.25	0.24	0.22	0.17
	τ_*2*_	796.62	67.82	162.08	499.74	962.46	835.03	1124.60	270.23
	g3		0.22	0.18					
	τ_*3*_		794.17	1241.8					
Ogden N1	μ_1_	0.003	0.22	0.03	0.02	0.05	0.01	0.05	0.38
	α_1_	22.46	−4.45	8.93	20.57	11.37	16.39	14.88	6.35
Neo Hookean	c10	0.006	0.09	0.02	0.05	0.05	0.006	0.04	0.25
Yeoh	c10	0.007	0.14	0.02	−0.05	0.03	0.005	0.02	0.25
	c20	−0.07	−0.38	−0.06	0.66	−0.02	0.10	0.53	−0.42
	c30	0.52	0.58	0.34	−0.60	0.60	−0.06	−0.85	2.48

### Cyclic Tests

**FIGURE 9:**
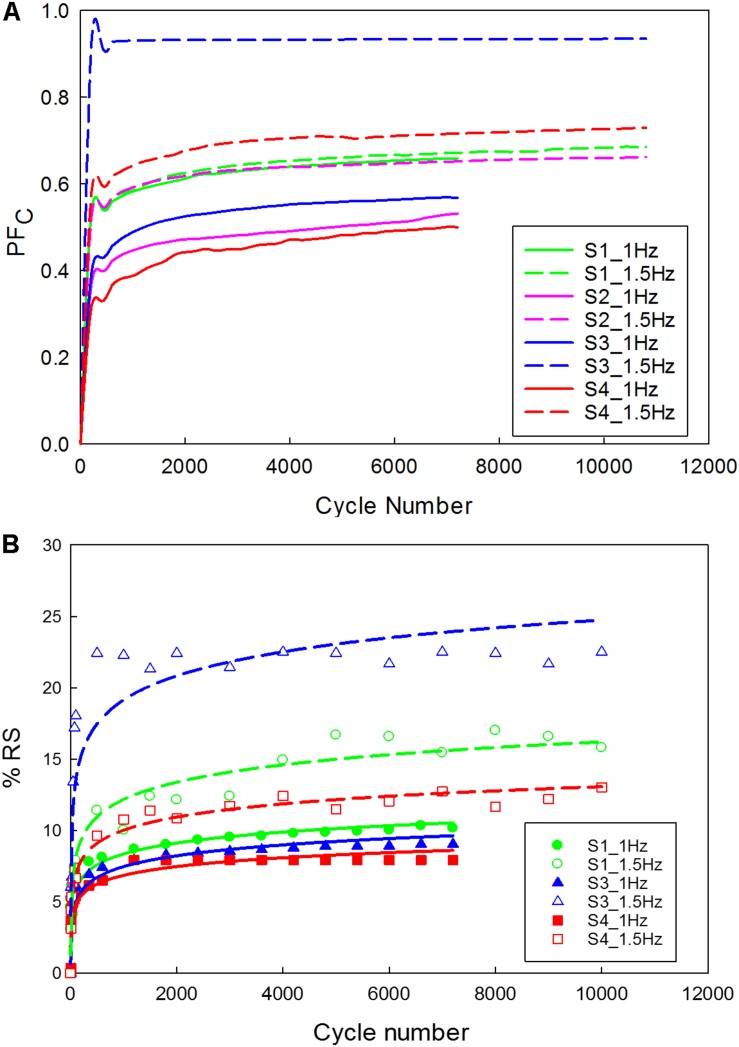
Carotid plaque tissue was subjected to 30% stretch at 0.1 mm/s, followed by a sinusoidal waveform (amplitude corresponding to 20% stretch) of 1 Hz and 1.5 Hz frequencies for 2 h. **(A)** Normalized variation in peak force as a function of time; **(B)** Residual strain accumulation as a function of cycle number (The function used for logarithmic curve fitting is *y* = a × ln(x) + b).

**FIGURE 10:**
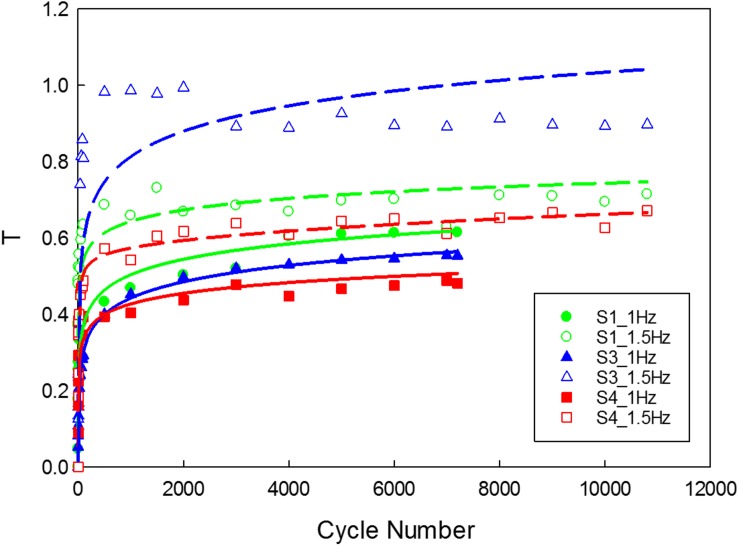
Variation in the tangent modulus of the unloading curve of the tissue. (The function used for logarithmic curve fitting is *y* = a × ln(x) + b).

**FIGURE 11:**
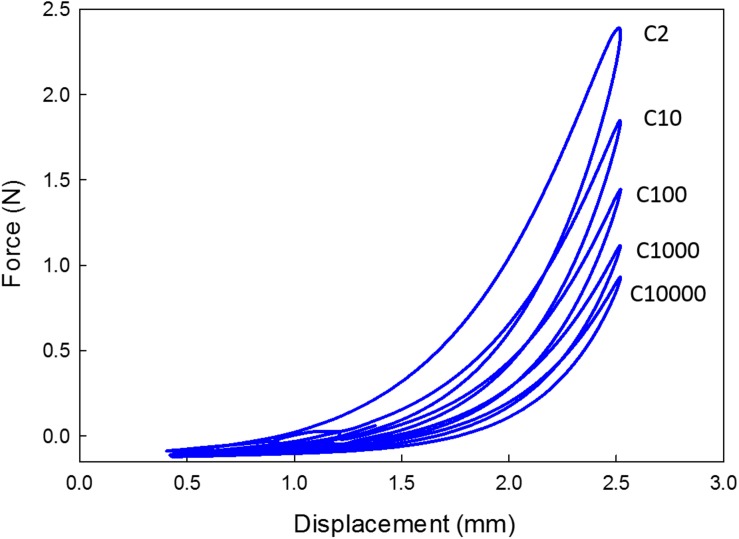
Hysteresis loops of selected cycles for a 1.5 Hz frequency profile (C- Cycle number).

The data corresponding to the dimension, width to gauge length ratio and type of the strip is listed in Table 3. The normalized variation in the peak force of any cycle n with respect to cycle one was calculated and represented in Figure 9A. During the loading phase of each cycle, zero forces were recorded for the first few millimeters of the displacement, and this trend increased with increase in cycle number. Therefore, to quantify the residual strain accumulation throughout the test, the maximum displacement corresponding to the zero force for any cycle *n* with respect to cycle 2 was determined. The percentage of the residual strain accumulation was plotted in Figure 9B. Also, to observe the changes in the mechanical behavior of the samples, the tangent modulus of the unloading curve of any cycle *n* with respect to cycle one was calculated (Figure 10). A continuous change in peak force and strain accumulation was observed during the cyclic test. This data demonstrates the fatigue behavior and shows that most of the softening occurs in the initial cycle. Figure 11 illustrates the results obtained from a cyclic test (selected cycles are represented). It was observed that the force during sinusoidal loading dipped below 0 N and was apparent for all samples tested. This observation may be due to the rate of fluctuation being faster than the elastic recoil ability of the carotid tissue.

## Discussion

From a biomechanical standpoint, arteries are viscoelastic, non-linear, and anisotropic (Fung, 1967; Holzapfel et al., 2004; van Dam et al., 2008). They exhibit time-dependent properties and are subjected to pulsatile hemodynamic loads. Hence, understanding the mechanical response of the plaque tissue under stress-relaxation and cyclic loading conditions is vital. Therefore, this study focused on the characterization of the time-dependent properties of the carotid plaque tissue by mimicking the *in vivo* conditions. Uniaxial tensile and control tests were also performed to determine the Cauchy stress and relative stiffness of the tissue samples over time.

### Uniaxial Tensile Tests and Control Tests

The Cauchy stress of the 16 strips cut from the plaque tissue exhibited considerable variation between samples acquired from different patients and within the same patient. This variation in the stress was due to uneven specimen thickness and difference in the morphology of the plaque tissue at different locations. Stress concentration exists near the fixtures. Initially, stress increased slowly with an increase in the stretch ratio, followed by a rapid rise before failure occurred. Moreover, an initial drop in the load-displacement curve indicated early damage of the tissue sample. Also, the mean stress and stretch values for the samples with calcification was found to be lower than the strips with no-calcification. The abrupt variation in stress values might be attributed to the separation of calcification and the surrounding tissue causing damage to the sample due to the presence of stress concentrations at the interfaces (Abedin et al., 2004). Previous studies have also similarly suggested that stresses acting on the tissue depends on the density of calcification and the interaction between the calcification and surrounding tissue (Barrett et al., 2016a, 2017).

The stress values at failure varied from 270 kPa to 2.3 MPa. The stretch ratio ranged from 1.4 to 2.95. The variation in stress and stretch values may be due to the variation in the pathology, width of the samples, and the thickness along the length of the sample (Table 2). The non-calcified strips (soft) have lower Cauchy stress values at failure in comparison to the strips which are partially calcified and calcified strips (see Supplementary Figure S3). Also, the Cauchy stress and stretch values at failure could have been influenced by the density and relative position of calcification in the surrounding tissue (Hoshino et al., 2009; O’Leary et al., 2015). Since the strip components may have a mixture of soft lipid core, calcification, and fibrous tissue; presence of soft lipid reduces the stability of the plaque (Lendon et al., 1991; Grønholdt et al., 2002). Control tests were performed to investigate the effect of test duration. The tissue exhibited a 8.7% decrease in the elastic properties of the tissue within 6 h (Gasser et al., 2008). The maximum time of the sample being tested was 2 h for the cyclic tests, where the decrease in the elastic properties of the tissue is less than 3%.

### Stress-Relaxation and Cyclic Tests

Viscoelastic behavior of the plaque tissue was explained by *F*_SR_(*t*) versus the time curve represented in Figure 7. This figure shows that the normalized relaxation force increased initially and tended to stabilize toward the end of relaxation phase. The curves of 0.1 mm/s, 1 mm/s strain rates seemed to be parallel to each other and indicated stabilization. The variability of the normalized force between different strain rates was minimum for the samples with high calcification compared to the samples with medium and low calcification. Also, the normalized relaxation force represented by the blue curve obtained at 1 mm/s is lower than the one for 0.1 mm/sec. This strip and sample have high calcification content (hard). During testing there has been a separation of the calcification region from the surrounding tissue. There might also be micro damage in the tissue that resulted in lower values in comparison to 0.1 mm/sec. Histological examinations of the sample before and after testing are required to identify any accumulated damage or change in orientation of the collagen fibers. The stress-strain response of initial loading where a 30% stretch is applied at 0.1 or 1 mm/s exhibited non-linear behavior. The second part of the curve where the displacement was maintained constant highlights the viscoelastic behavior of the samples. Material constants listed in Table 2 were computed using the ABAQUS/Explicit 6.13. These material constants are consistent with findings in the literature and may assist in patient-specific vulnerability assessments when applied to finite element simulations (Lawlor et al., 2011; Heiland et al., 2013).

In the physiological state, carotid plaque tissue is in a stressed state, hence during the cyclic test, the samples were stretched up to 30% of the gauge length, and then a 20% sinusoidal stretch was applied. The cyclic test protocol displayed apparent stabilization phenomena in the peak reaction force as a function of cycle number Figure 8A. The hysteresis loop decreased and became narrow as a function of cycle number. This finding is in alignment with previous literature (Fung, 1967; Pasquesi et al., 2016), that reported similar phenomena of various biological soft tissues under different strain rates. The initial cycle exhibited a large amount of softening, and exponential decrease of the hysterics loop with successive cycling due to the reorientation or damage of collagen fibers (Pasquesi et al., 2016).

The peak force recorded for strips tested under 1.5 Hz frequency was higher when compared to strips tested under 1 Hz frequency. The high cyclic frequency *in vivo*, which is one of the risk factors for cardiovascular diseases is crucial in disease management. This is attributed to the increase in plaque stresses and strains leading to progression of the disease that may result in acute events (Brankovic et al., 2013; Tang et al., 2017). Similarly, other underlying mechanisms which may be attributed to stabilization include fatigue softening. During the initial cyclic loading, there was a continuous increase in peak force, which was followed by a sudden decrease before stabilization. Though all the samples exhibited a similar behavior, different peak forces were recorded. The difference in peak forces may be due to the difference in the disease state of the patient (see Supplementary Table S1) and variation between samples due to the different components (Table 2).

As the cycle number increased, the residual strain of the sample also increased (Pasquesi et al., 2016; Remache et al., 2018). For the samples tested with 1 Hz frequency (Figure 9B), the residual strain increased by about 8% comparing to the 1.5 Hz frequency which increased by about 20%. This indicates the frequency dependent behavior of the tissue (Pasquesi and Margulies, 2017). Similarly, the variation in tangent modulus (Figure 10) increased with the cycle number, indicating alteration of the mechanical properties due to repeated cyclic loading. Also, the higher the frequency of cyclic loading, the higher the accumulation of damage. The strips with a higher calcification (hard) exhibited a greater change in mechanical properties (Barrett et al., 2017).

Displacement controlled loading protocols were used in the present study. The amplitude of the waveform (displacement) was extracted from the initial load level based on the uniaxial tensile tests. However, patient-specific pulsatile loading exists *in vivo*. The amplitude and shape of the waveform vary with time, and risk factors. Also, the hemodynamic forces, biological and chemical changes that take place influence the waveform in one cardiac cycle. In this study samples subjected to displacement-controlled protocol exhibited stress-relaxation over time thereby reducing the stress levels that are too small to initialize failure. But clinically stresses depend on the hemodynamic forces that are related to the risk factors and the material’s response to the forces acting on it. Moreover, it is evident that the behavior of conventional materials depends on the shape and amplitude of the waveform. However, the data presented in this study using displacement-controlled protocols can be used to understand the material behavior of the plaque under different loading conditions and can have clinical relevance when the effect of wave form is considered while interpreting the results. The data presented in this study also supports the idea that treatments should be tailored based on the composition of the plaque.

The mechanical behavior of the healthy and diseased tissue is very different and depends on the disease severity and patient-specific risk factors including modifiable and unmodifiable risk factors. To understand the disease progression and provide accurate treatment procedures, it is important to understand the mechanical behavior and change in material properties under the dynamic environment of the cardiovascular system. The material response in association with risk factors may help clinicians to predict the severity of the disease and tailor patient-specific treatment procedures. This preliminary study provided first experimental data to better understand the plaque mechanical behavior under cyclic loading conditions. To achieve a significant conclusion on the mechanical properties of the plaque components further extensive studies on a larger number of tissue samples will be required. This study provided basic understanding of the differences in plaque properties with and without calcification and the stress-relaxation and cyclic behavior of the carotid plaque tissue. The experimental protocols developed in this study will be used in future studies to develop association between plaque property and vulnerability. Future long-term failure studies under pulsatile loading in combination with specimen-specific finite element studies will improve our understanding of plaque mechanical behavior under different loading conditions.

### Limitations

There are limitations associated with this preliminary study which need to be noted. First, the plaque mechanical properties were investigated in the circumferential direction, while multi-axial loading exists *in vivo*. Second, there were apparent irregularities in the sample dimensions. To account for this, normalized values were reported. However, a possible solution to overcome the challenge of irregular specimens is to use specimen-specific finite element models (Zhu et al., 2010; Aldieri et al., 2018). Third, this study used simple cyclic loading while pulsatile loading exists *in vivo.* Future studies should consider loading protocols resembling the pulsatile loading. Fourth, no histological analysis was performed to study the change in the damage accumulation of the tissue before and after the testing. The changes in the orientation of the collagen fibers before and after mechanical testing will provide an understanding of how tissue responds to different loading types. Fifth, components present may have induced stresses in the samples. Sixth, a displacement-controlled testing protocol was used for the experiments. Instead, a load-controlled testing protocol may be applicable to investigate the cyclic behavior of the viscoelastic materials as *in vivo* loading conditions include pressure load due to blood flow in the arteries. Also, the stress level at high load cycles is too small to initialize failure when intended to determine fatigue failure properties. Therefore, to study the fatigue behavior load-controlled testing protocol is more appropriate in comparison to displacement-controlled testing protocol. Future studies should consider force-controlled testing protocols based on the cardiac cycle. Seventh, viscous behavior of the sample during the first 30% extension for 1 mm/sec deformation rate was not considered while extracting the material properties. Eighth, a relatively small sample size and loading conditions were tested. Therefore, quantifying the statistical differences has been a challenge due to relatively low number of samples and different number of strips tested from each patient. Therefore, future studies should consider a large number of samples with uniform strips across patients. As the aim of the study was to analyze the effect of repeated loading on the carotid plaque sample, preconditioning was not considered. For the validity and clinical relevance of the data more samples with multiaxial pulsatile loading on a whole plaque tissue will be more relevant. Future large-scale studies with different loading conditions are necessary to capture the full range of time-dependent mechanical properties of the carotid plaque tissue. Likewise, load-controlled prolonged cyclic loading until failure may assist in the characterization of the failure properties of the plaque tissue.

## Conclusion

Carotid plaque tissue samples were subjected to uniaxial tensile test, control test, stress-relaxation test and cyclic loading test protocols. The time-dependent mechanical response of the carotid plaque tissue was investigated by applying cyclic loading under physiological temperature and hydration medium. The Cauchy stress values of the tissue samples varied with calcification. The normalized relaxation force due to stress-relaxation test increased initially and stabilized toward the end of stress- relaxation phase, highlighting viscoelastic behavior. During the cyclic tests, there was a decrease in the peak force as a function of cycle number indicating mechanical distension due to repeated loading. The tissue also accumulated residual deformation as a function of cycle number, which was attributed to fatigue softening. Despite the variations in the samples, the overall trends were similar. This work represents a step toward an improved understanding of the material behavior of the human atherosclerotic plaques. Further study on the fatigue failure of the carotid plaque tissue will be required to determine failure properties that may assist in the plaque vulnerability assessment.

## Data Availability Statement

The datasets generated for this study are available on request to the corresponding author.

## Ethics Statement

This study was approved by the Human Research Ethics Committee at the Princess Alexandra Hospital (PAH) in Brisbane, Australia, and QUT’s Office of Research Ethics and Integrity (HREC/17/QPAH/181). All procedures performed in the studies were in accordance with the ethical standards of the institutional and/or a national research committee. Informed consent was obtained from all individual participants included in the study.

## Author Contributions

PP, PY, YG, and ZL designed the work. PP and RK conducted the experiments. PP, JW, and JM analyzed the results. TM and TL provided their expertise in MRI imaging and vascular mechanics. PP wrote the manuscript text. All authors reviewed the manuscript.

## Conflict of Interest

The authors declare that the research was conducted in the absence of any commercial or financial relationships that could be construed as a potential conflict of interest.

## Footnotes

1 imagej.nih.gov/ij/
