# Supplementary material for: Stress-Relaxation and Cyclic Behavior of Human Carotid Plaque Tissue
Source: Front Bioeng Biotechnol. 2020 Feb 11;8:60. doi: 10.3389/fbioe.2020.00060 (PMC7026010; doi:10.3389/fbioe.2020.00060)
Supplement: Supplementary file 1 [file Data_Sheet_1.docx]

**Supplementary Material**

**Introduction**

This supplementary material provides information regarding the testing profiles, patient demographics, measurement of the %area of calcification, Cauchy stress and stress ratio for different tensile test samples.

Table I presents information relating to the patient demographics and disease state of the samples used for tensile testing (TI, TII, TIII, and TIV), stress-relaxation and cyclic fatigue test (S1, S2, S3, and S4)

Table II presents information relating to the testing profiles used for the stress-relaxation test (different strain rates)

Table III presents information relating to the testing profiles used for the cyclic test (different frequency of cyclic loading)

Figure I present information about the stress relaxation used to identify the material constants

Figure II presents information regarding the % area calcification from a low energy x-ray using Image J.

Figure III presents information about the Cauchy stress and stretch ratio of the tensile test strips. The strips are characterized as soft, mixture and hard based on the components present in the strip.

Figure IV presents the Cauchy stress-stretch ratio curves for all the tensile test specimens

*Table I: Patient demographic data of the investigated carotid plaque samples*

| ID | TI | TII | TIII | TIV | S1 | S2 | S3 | S4 |
| --- | --- | --- | --- | --- | --- | --- | --- | --- |
| Gender | M | M | F | F | M | F | M | M |
| Age(Years) | 53 | 79 | 72 | 63 | 66 | 76 | 70 | 70 |
| Stenosis (%) | 80-99 | 70-80 | 50-79 | 80-90 | 50-79 | 80-99 | 50-70 | 50-80 |
| Blood Pressure | 157/84 | 159/63 | 165/81 | 135/82 | 130/78 | 135/91 | 170/53 | 157/81 |
| Weight (kg) | 104 | 61 | 97 | 92 | 103 | 77 | 66 | 84 |
| Hypertension | Y | Y | Y | Y | Y | Y | Y | Y |
| Hypercholesteremia | Y | Y | Y | Y | Y | Y | Y | Y |
| Diabetes | N | N | Type 2 | N | N | N | N | Type 2 |
| Smoking | Former | Former | N | Former | Former | Current | Current | Current |
| Calcification | High | Low | Medium | Medium | Low | Low | High | Medium |

Table II: Stress-relaxation Test Profile 1 and Profile 2

| Profile | Command | Mode | Position (mm) | Rate (mm/s) | Time (hours) |
| --- | --- | --- | --- | --- | --- |
| 1 | Displacement | Relative Ramp | 0 – 1.5 | 0.1 | - |
|  | Hold | Hold | 1.5 | - | 1 |
| 2 | Displacement | Relative Ramp | 0 – 1.5 | 1 | - |
|  | Hold | Hold | 1.5 | - | 1 |

Table III: Cyclic Fatigue Test Profile 1 and Profile 2

| Profile | Command | Mode | Position (mm) | Rate (mm/s) | Frequency (Hz) | Time (hours) |
| --- | --- | --- | --- | --- | --- | --- |
| 1 | Displacement | Relative Ramp | 0 – 1.5 | 0.1 mm/s | - | - |
|  | Waveform | Sinusoidal | ±1 | - | 1 | 2 |
| 2 | Displacement | Relative Ramp | 0 – 1.5 | 0.1 mm/s | - | - |
|  | Waveform | Sinusoidal | ±1 | - | 1.5 | 2 |


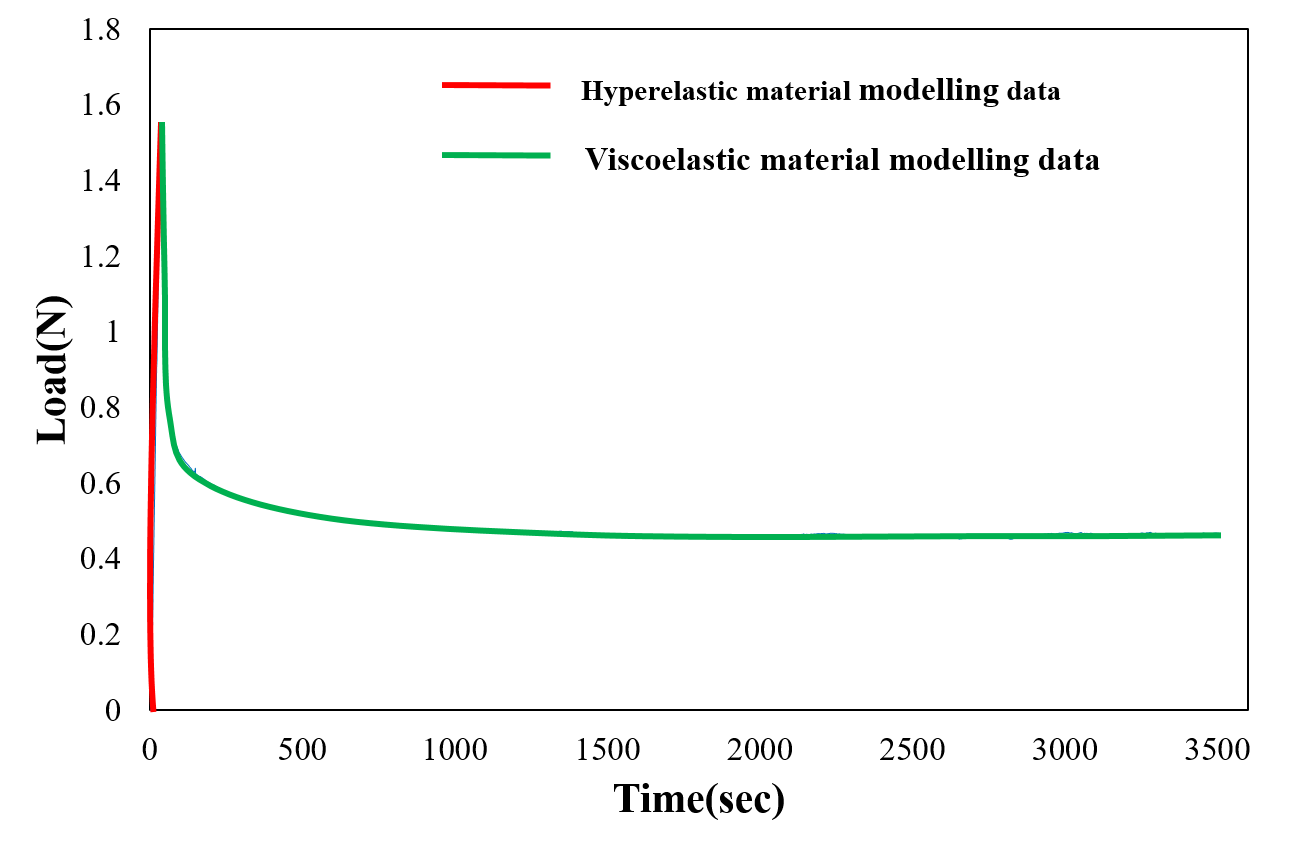


Figure I: Representation of the stress-relaxation data used for material modelling. Red Curve was used to calibrate the material constants for Neo-Hookean, Ogden (order 1) and Yeoh models; Green curve was used calibrate the viscoelastic material behaviour


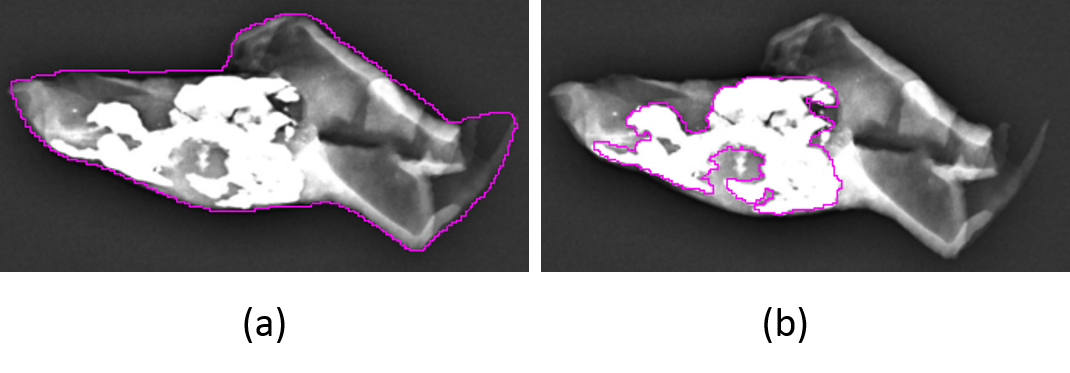


Figure II: Quantification of % area calcification from a low energy x-ray image

Figure III: Cauchy stress and stretch ratio for different tensile test strips

Figure IV: Stress-stretch ratio curves of tensile tests for different strips tested
